# Hemokinin-1 Gene Expression Is Upregulated in Trigeminal Ganglia in an Inflammatory Orofacial Pain Model: Potential Role in Peripheral Sensitization

**DOI:** 10.3390/ijms21082938

**Published:** 2020-04-22

**Authors:** Timea Aczél, Angéla Kecskés, József Kun, Kálmán Szenthe, Ferenc Bánáti, Susan Szathmary, Róbert Herczeg, Péter Urbán, Attila Gyenesei, Balázs Gaszner, Zsuzsanna Helyes, Kata Bölcskei

**Affiliations:** 1 Department of Pharmacology and Pharmacotherapy, Medical School & Szentágothai Research Centre, Molecular Pharmacology Research Group, Centre for Neuroscience, University of Pécs, H-7624 Pécs, Hungary; aczel.timea@pte.hu (T.A.); angela.kecskes@aok.pte.hu (A.K.); kun.jozsef@pte.hu (J.K.); zsuzsanna.helyes@aok.pte.hu (Z.H.); 2 Szentágothai Research Centre, Bioinformatics Research Group, Genomics and Bioinformatics Core Facility, University of Pécs, H-7624 Pécs, Hungary; herczeg.robert@pte.hu (R.H.); urban.peter@pte.hu (P.U.); gyenesei.attila@pte.hu (A.G.); 3 Carlsbad Research Organization Ltd, H-9244 Újrónafő, Hungary; kszenthe@carlsbad.hu; 4 RT-Europe Ltd, H-9200 Mosonmagyaróvár, Hungary; fbanati@rt-europe.org; 5 Galenbio Ltd, H-9200 Mosonmagyaróvár, Hungary; sszathmary@galenbio.com; 6 Department of Anatomy, University of Pécs Medical School, H-7624 Pécs, Hungary; balazs.b.gaszner@aok.pte.hu; 7 PharmInVivo Ltd., H-7629 Pécs, Hungary

**Keywords:** orofacial pain, hemokinin-1, trigeminal ganglia, complete Freund’s adjuvant, macrophages, satellite glial cells, neuroinflammation

## Abstract

A large percentage of primary sensory neurons in the trigeminal ganglia (TG) contain neuropeptides such as tachykinins or calcitonin gene-related peptide. Neuropeptides released from the central terminals of primary afferents sensitize the secondary nociceptive neurons in the trigeminal nucleus caudalis (TNC), but also activate glial cells contributing to neuroinflammation and consequent sensitization in chronic orofacial pain and migraine. In the present study, we investigated the newest member of the tachykinin family, hemokinin-1 (HK-1) encoded by the *Tac4* gene in the trigeminal system. HK-1 had been shown to participate in inflammation and hyperalgesia in various models, but its role has not been investigated in orofacial pain or headache. In the complete Freund’s adjuvant (CFA)-induced inflammatory orofacial pain model, we showed that *Tac4* expression increased in the TG in response to inflammation. Duration-dependent *Tac4* upregulation was associated with the extent of the facial allodynia. *Tac4* was detected in both TG neurons and satellite glial cells (SGC) by the ultrasensitive RNAscope in situ hybridization. We also compared gene expression changes of selected neuronal and glial sensitization and neuroinflammation markers between wild-type and *Tac4*-deficient (*Tac4^-/-^*) mice. Expression of the SGC/astrocyte marker in the TG and TNC was significantly lower in intact and saline/CFA-treated *Tac4^-/-^* mice. The procedural stress-related increase of the SGC/astrocyte marker was also strongly attenuated in *Tac4^-/^*^-^ mice. Analysis of TG samples with a mouse neuroinflammation panel of 770 genes revealed that regulation of microglia and cytotoxic cell-related genes were significantly different in saline-treated *Tac4^-/-^* mice compared to their wild-types. It is concluded that HK-1 may participate in neuron-glia interactions both under physiological and inflammatory conditions and mediate pain in the trigeminal system.

## 1. Introduction

Sensitization of trigeminal nociceptors by inflammatory mediators is a major factor of orofacial pain and headache disorders by causing hyperalgesia and allodynia. The orofacial area is mainly innervated by ophthalmic, maxillary and mandibular branches of the trigeminal nerve. The cell bodies of the primary sensory neurons are located in the trigeminal ganglia (TG) and their central terminals relay the protopathic information to the spinal trigeminal nucleus caudalis (TNC) and the upper cervical dorsal horn. When trigeminal nerve injury or orofacial inflammation occurs, TG neurons become sensitized and in turn release several mediators, which lead to enhanced responsiveness of the secondary afferent neurons. Satellite glial cells (SGC) and resident macrophages in the TG, as well as astrocytes and microglia in the central nervous system, are also activated in the process and contribute to the sensitization. There is growing evidence that the cross-talk between neurons and glial cells has a prominent modulatory role on nociceptive transmission under physiological and pathophysiological conditions [1,2,3,4,5,6]. 

A major percentage of nociceptive sensory neurons both in the trigeminal and dorsal root ganglia are peptidergic and release several neuropeptides, such as tachykinins (substance P (SP) and neurokinins) and calcitonin gene-related peptide (CGRP) in response to activation [7,8]. Tachykinins can be released both from the peripheral and central endings of primary sensory neurons contributing to inflammatory processes and pain transmission [9,10]. The biological actions of tachykinins are mediated by the G-protein coupled neurokinin NK-1, NK-2 and NK-3 receptors [11,12]. NK-1 receptor antagonists were shown to effectively reduce neuropathic mechanical hyperalgesia and inflammatory pain in animal models [13,14,15]. Since SP is expressed by trigeminal sensory neurons, it also became the focus of migraine studies. SP released from the peripheral terminals of trigeminal neurons induces plasma protein extravasation and vasodilatation in the dura mater [16], while centrally in the TNC it contributes to pain transmission [17,18]. It has been shown that the SP/NK-1 system participates in orofacial heat hyperalgesia in inflammatory and nerve injury-related animal models [19]. Preclinical data were promising regarding the use of NK-1 receptor antagonists in several pain conditions and inflammatory disease models [19,20,21]. Nevertheless, human studies could not prove the analgesic effect of these compounds, either in migraine [22,23,24,25] or in other conditions like post-operative dental pain [26] or neuropathic pain [27]. The explanation for the failure of NK-1 receptor antagonists as analgesics and anti-migraine drugs remains unclear, but it might be due to differences of the human NK-1 receptor structure and function as compared to the rodent receptor, or the ineffectiveness of competitively blocking the SP binding site [28,29]. 

The discovery of the newest member of the tachykinin family, the hemokinin-1 (HK-1) encoded by the *Tac4* gene [30], has given a new impetus to tachykinin research. HK-1 might be a novel key molecule in behavior, pain and inflammatory processes [31]. There is a growing amount of data regarding the mRNA expression of the *Tac4* gene both in the central and peripheral nervous systems. In contrast to other tachykinin members, relatively high expression of the *Tac4* gene can be found in the periphery (e.g., lung, spleen, adrenal gland). B and T lymphocytes, macrophages and dendritic cells also express *Tac4* [30,32,33,34]. While other tachykinins are conserved across mammals, HK-1 is highly homologous in mouse and rat, but not in humans. Both rodent peptides and the human HK-1 can bind to the NK-1 tachykinin receptor [35], but they also have distinct, NK-1-independent actions [36,37]. Since the structures of HK-1 and SP are very similar, it is difficult to proceed with peptide detection, localization and measurement. Although a few studies reported antibody development (e.g., [38]) or a recent immunohistochemical study on the TG with an in-house developed antibody [39], there are still no commercially available antibodies against HK-1. Despite the structural similarities and common receptors of HK-1 and SP, some of their functions appear to be different, even opposing each other [36,40,41,42,43]. This could be explained by different binding sites and different signalling pathways by HK-1 compared to SP even at the NK-1 receptor, but a specific target is suggested to mediate several actions of HK-1 [36]. Therefore, since the receptorial mechanisms of HK-1 are not precisely known, pharmacological interventions (e.g., antagonists) cannot be used to validate the target.

Studies investigating the pain modulatory function of HK-1 showed opposing effects, pointing to a complex role of the peptide. HK-1 had a pronociceptive effect after intrathecal or intracerebroventricular administration, causing pain and scratching behavior without influencing the withdrawal latency to a noxious heat stimulus [34,43]. However, in other studies, an analgesic effect was shown upon intracerebroventricular injection [44,45,46]. Its potential contribution to pain sensitization was shown by the upregulation of *Tac4* mRNA expression in lipopolysaccharide-stimulated cultured microglia [47], and in the rat spinal dorsal horn after complete Freund’s adjuvant (CFA)-induced paw inflammation [48]. 

The role of HK-1 in orofacial pain and trigeminal sensitization have been poorly investigated. Therefore, we aimed to explore the potential role of HK-1 in the trigeminovascular system by (i) detecting expression changes of *Tac4* in the TG and TNC in a rat inflammatory orofacial pain model [49] (ii) investigating behavioral alterations and gene expression changes of selected markers of neuronal sensitization and neuroinflammation by comparing *Tac4*-deficient (*Tac4^-/-^*) and wild-type mice.

## 2. Results

### 2.1. Tac4 mRNA Levels Are Upregulated in Response to CFA-Induced Inflammation in the Rat TG in Association with Facial Allodynia

**Figure 1:**
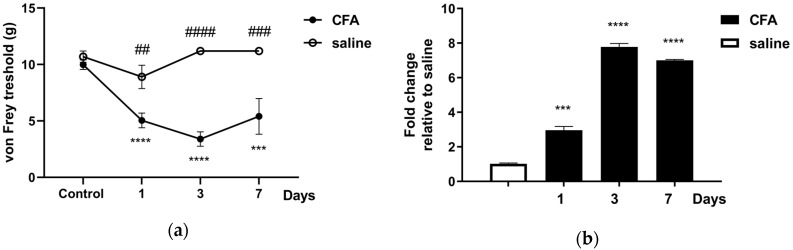
(**a**) Changes in mechanical threshold measured with von Frey filaments on day 1, 3, 7 after unilateral injection of complete Freund’s adjuvant (CFA) or saline (50 μL s.c.). Data are means ± S.E.M. (CFA: *n* = 5–16, saline: *n* = 6). A sterisks denote statistically significant differences between the control day and the days after CFA treatment (*** *p* ≤ 0.001, **** *p* ≤ 0.0001), while hash marks label statistically significant differences between saline and CFA groups (## *p* ≤ 0.01, ### *p* ≤ 0.001, #### *p* ≤ 0.0001) as analyzed by two-way ANOVA followed by Tukey’s multiple comparison tests; (**b**) time course of normalized fold changes in *Tac4* mRNA expression of rat trigeminal ganglia (TG) on day 1, 3 and 7 after CFA or saline injection. The mRNA levels were normalized to *β2m* and *Hprt1*. Data are means ± S.E.M. (*n* = 3 at each time point). Asterisks denote statistically significant differences between saline and CFA groups (*** *p* ≤ 0.001, **** *p* ≤ 0.0001), as analyzed by one-way ANOVA followed by Tukey’s multiple comparison tests.

The facial mechanonociceptive threshold of CFA-injected rats was significantly decreased compared to saline injection, starting from day one until day seven after injection. The allodynia reached its maximum on day three in the whisker pad area (Figure 1a), in accordance with our previous results [49]. *Tac4* mRNA expression was also measured in peripheral blood mononuclear cells (PBMCs), TG and TNC tissues. The fold changes of *Tac4* mRNA in TG (Figure 1b) followed the course of von Frey threshold changes, reaching its maximum on day three. In TNC and PBMC samples the *Tac4* expression could not be detected with sufficient reliability with this method, as C_q_ values were very close to the detection limit.

### 2.2. CFA-Induced Orofacial Inflammation Upregulates Tac4 mRNA in Both Primary Sensory Neurons and SGCs of the Rat TG

**Figure 2:**
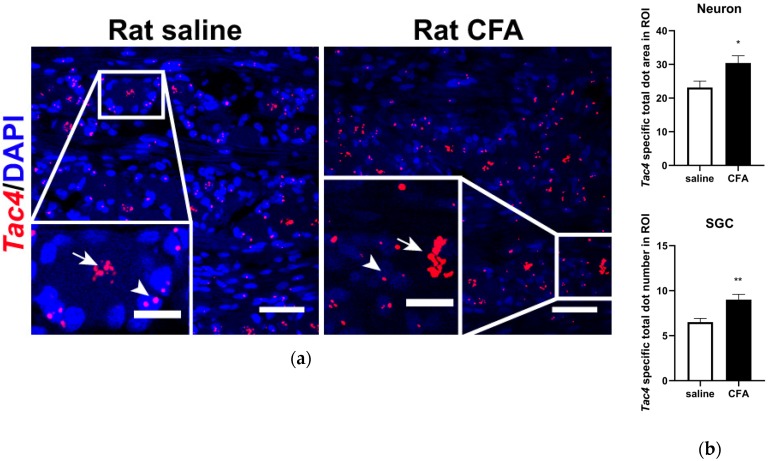
(**a**) Representative confocal images of *Tac4* mRNA (red) counterstained with DAPI are shown on longitudinal slices of rat TG after injection of saline or CFA. Arrows indicate sensory neurons, arrowheads refer to SGCs. Scale bar: 50 µm, inset scale bar: 20 µm; (**b**) quantification of *Tac4* mRNA showing upregulation after CFA compared to saline injection in *Tac4*-positive neurons and SGCs of *n* = 4 (saline)-6 (CFA) rats/group. Asterisks denote statistically significant differences between saline and CFA groups (* *p* ≤ 0.05, ** *p* ≤ 0.01), as analyzed by Student’s *t*-test for unpaired samples. ROI: region of interest; unit of the area: μm^2^.

To investigate the basal expression and inflammation-induced alterations of the *Tac4* mRNA in the rat TG, fluorescent RNAscope in situ hybridization (ISH) was performed, that provides cellular resolution and tissue context. *Tac4* transcripts were localized primarily on sensory neurons and SGCs in saline-treated samples (Figure 2a, left panel) and significantly upregulated in both cell types upon CFA treatment (Figure 2a, right panel). Basal and elevated *Tac4* mRNA levels were analyzed semi-quantitatively using ImageJ software and plotted as *Tac4*-specific total dot area/number detected in sensory neuron soma or SGC (Figure 2b). Sensory neurons and SGCs were identified morphologically (see arrows and arrowheads, respectively, Figure 2a) and histologically by colocalizing *Tac4* with neuronal (NeuN, encoded by *Rbfox3*) and satellite glial marker (SK3, encoded by *Kcnn3*, see Supplementary Figure S2). RNAscope performed on rat TG was validated by RNAscope 3-plex negative control probes designed to bacterial *dapB* gene giving no detectable fluorescent signal on any channel (Supplementary Figure S3a,b). RNAscope 3-plex mouse positive control probes were used to visualize the housekeeping genes: RNA polymerase II subunit A (*Polr2a*), peptidyl-prolyl cis-trans isomerase B (*Ppib*) and polyubiquitin-C (*Ubc*) mRNA on rat TG from saline-treated animals (Supplementary Figure S3c,d). 

### 2.3. CFA-Induced Orofacial Inflammation Upregulates Tac4 mRNA in Both Primary Sensory Neurons and SGCs of the Mouse TG

**Figure 3:**
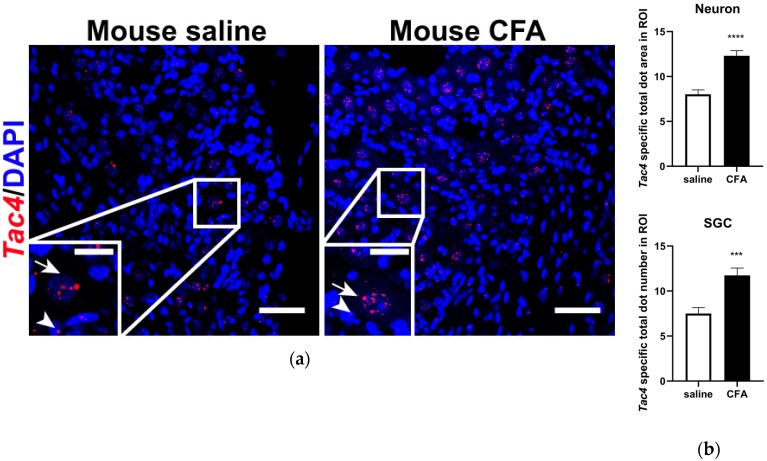
(**a**) Representative confocal images of *Tac4* mRNA (red) counterstained with DAPI is shown on longitudinal sections of mouse TG after saline or CFA treatment. Arrows indicate sensory neurons, arrowheads refer to SGCs. Scale bar: 50 µm, inset scale bar: 20 µm; (**b**) statistics showing *Tac4* mRNA upregulation after CFA compared to saline injection in *Tac4*-positive neurons and SGCs of *n* = 4 (saline) -5 (CFA) mice/group. Asterisks denote statistically significant differences between saline and CFA groups (*** *p* ≤ 0.001, **** *p* ≤ 0.0001), as analyzed by Student’s *t*-test for unpaired samples. ROI: region of interest; unit of the area: μm^2^.

Similarly to *Tac4* expression found in rat TG, basal *Tac4* mRNA was detected both in sensory neurons and SGCs of the mouse TG (Figure 3a, left panel). Also, mouse *Tac4* mRNA was shown to be upregulated in response to CFA-induced inflammation (Figure 3a, right panel). Sensory neurons and SGCs were identified morphologically (see arrows and arrowheads, respectively, Figure 3a). Technical control conditions using 3-plex negative (Supplementary Figure S3c) and mouse 3-plex positive (Supplementary Figure S3d) control probes were applied on longitudinal mouse TG from saline-injected animals.

### 2.4. CFA-Induced Alterations of Neuronal and Glial Activation Markers in the TG and TNC of Tac4 Gene-Deficient Mice

Mouse TG samples exhibited a relatively low value of *Tac4* expression levels, thus it could not be reliably detected and evaluated by RT-qPCR. We were unable to evaluate *Tac4* expression in TNC and PBMC samples either, similarly to rat TNC and PBMC samples.

**Figure 4:**
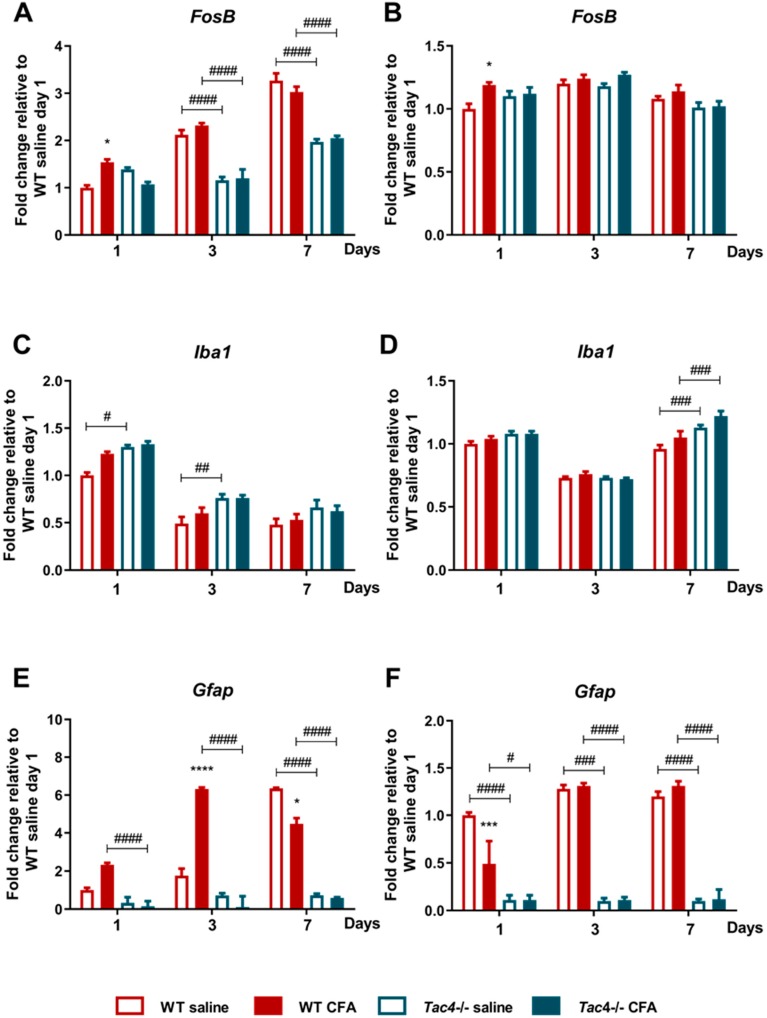
Time course of normalized fold changes in *FosB* (**A**,**B**), *Iba1* (**C**,**D**), *Gfap* (**E**,**F**) mRNA expression in the trigeminal ganglia (**A**,**C**,**D**) and trigeminal nucleus caudalis (**B**,**D**,**F**) of wild-type (WT) and *Tac4^-/-^* mice one, 3 and 7 days after saline/CFA injection. The mRNA levels were normalized to *Ppia* (TG) and *Ppia*, *Gapdh* (TNC), as detailed in Materials and methods. Data are means ± S.E.M. (WT CFA: *n* = 3–10, WT saline: *n* = 4–9, *Tac4^-/-^* CFA: *n* = 5–7, *Tac4^-/-^* saline: *n* = 5–10). Asterisks denote statistically significant differences between saline and CFA treated groups (* *p* ≤ 0.05, *** *p* ≤ 0.001), hash marks label statistically significant differences between WT and *Tac4^-/-^* groups (# *p* ≤ 0.05, ## *p* ≤ 0.01, ### *p* ≤ 0.001, #### *p* ≤ 0.0001), as analyzed by two-way ANOVA followed by Tukey’s multiple comparison tests.

We have previously described the gene activity profile of neuronal and glial activation markers in the rat TG and TNC, therefore we investigated the same markers in the mouse samples after orofacial inflammation. In wild-type (WT) mice, neuronal *FosB* gene expression was significantly upregulated by day 3 compared to intact samples (data not shown). However, not only CFA injection but also saline treatment caused an elevation of *FosB* in TG, therefore the differences between respective saline- and CFA-treated groups were not significant by days 3 and 7. A significant upregulation of the neuronal activation marker was only detected in *Tac4^-/-^* animals at a later time point, in day 7 samples. Comparison of matching WT and *Tac4^-/-^* animal groups showed that upregulation of the neuronal activation marker on days 3 and 7 was significantly lower in the TG of *Tac4^-/-^* mice (Figure 4a). In TNC only minor differences were seen (Figure 4b). 

In both TG (relative fold change WT: 1.00 ± 0.03 vs. *Tac4^-/-^*: 0.49 ± 0.03, *p* < 0.0001) and TNC (relative fold change WT: 1.00 ± 0.03 vs. *Tac4^-/-^*: 0.82 ± 0.02, *p* < 0.001) samples of intact animals, the microglia/macrophage activation marker (*Iba1*) showed significantly lower expression levels in *Tac4^-/-^* mice compared to WTs. *Iba1* expression was slightly higher in both saline- and CFA-treated *Tac4^-/-^* mice compared to their corresponding WT groups, being significant on day 1 and 3 in the TG and day 7 in the TNC. However, these differences were probably too small to be biologically meaningful (Figure 4c,d). The SGC/astrocyte activation marker *Gfap* was also expressed at a significantly lower level in the TG (relative fold change WT: 1.00 ± 0.16 vs. *Tac4^-/-^*: 0.33 ± 0.06, *p* < 0.01) and TNC (relative fold change WT: 1.00 ± 0.03 vs. *Tac4^-/-^*: 0.16 ± 0.05, *p* < 0.0001) of intact *Tac4^-/-^* compared to WT animals. After treatment, *Gfap* expression increased in all groups on all days compared to an intact group (data not shown). The effect of CFA on *Gfap* gene expression was a significant elevation in WT compared to the respective saline-treated group on day 1 and 3, but not in TNC samples. Moreover, *Gfap* levels were not altered due to CFA treatment in *Tac4^-/-^* mice. *Gfap* mRNA was decreased in all *Tac4^-/-^* groups compared to the WT group in most of the comparisons, both in TG and TNC (Figure 4e,f).

### 2.5. Neuroinflammation-Related Genes Are Differently Regulated between Saline-or CFA-Treated Tac4^-/-^ and WT Mice

**Figure 5:**
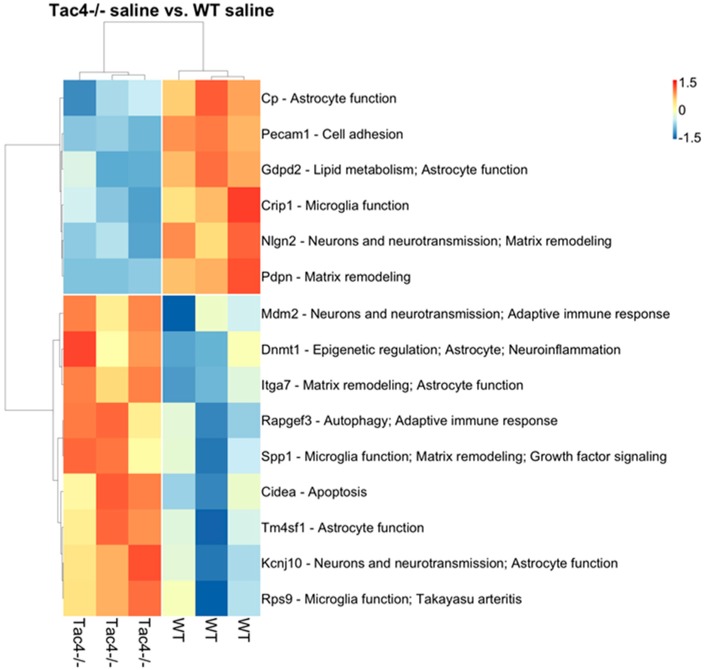
Heat map representation of the differentially expressed genes with annotations between TG samples of saline-treated *Tac4^-/-^* and WT mice. Rows represent genes and columns represent TG samples (*n* = 3 in each group). Normalized gene counts data are shown as row-wise z-scores (scale is shown on legend). Rows and columns were hierarchically clustered using Pearson correlation distance measure and average method. Distances are shown as dendrograms. References for the functional annotations: *Cp* [50] *Pecam1* [51] *Gdpd2* [52] *Crip1* [53] *Nlgn2* [54] *Pdpn* [55] *Mdm2* [56] *Dnmt1* [57] *Itga7* [58] *Rapgef3* [59] *Spp1* [60] *Cidea* [61] *Tm4sf1* [62] *Kcnj10* [63] *Rps9* [63].

**Figure 6:**
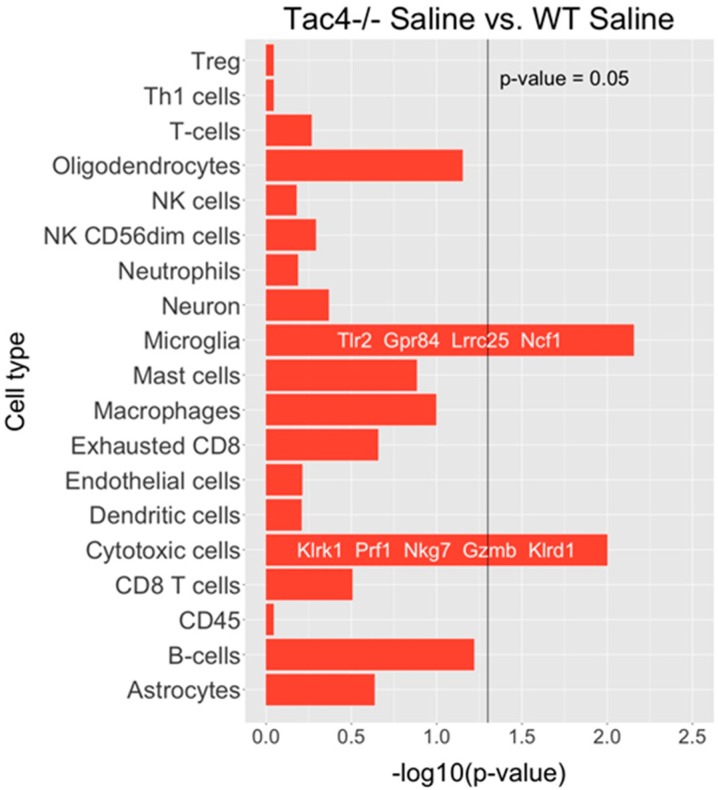
Barplots of *p*-values for correlation of cell-type-specific gene expressions as compared between saline-treated *Tac4^-/-^* and WT mice. *p*-values are -log_10_ transformed.

**Figure 7:**
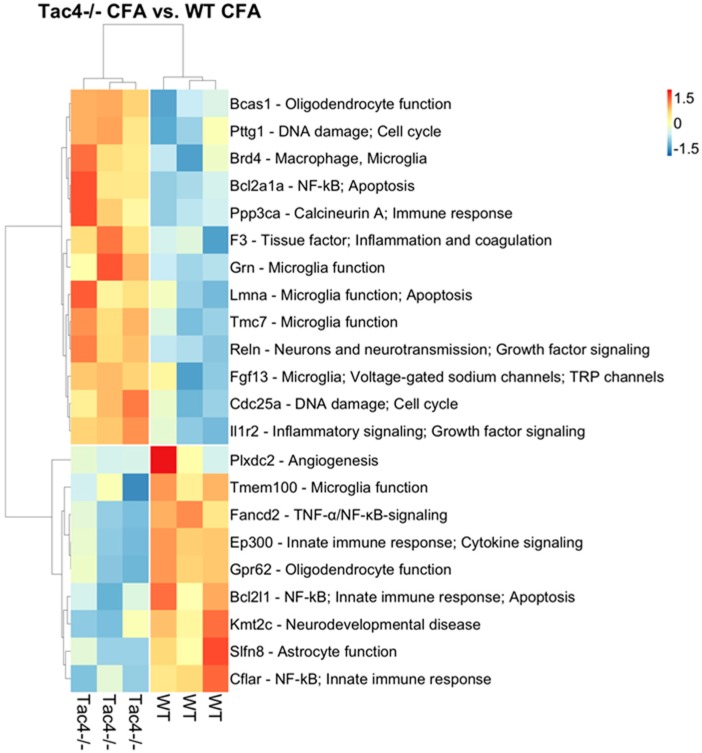
Heat map representation of the differentially expressed genes with annotations between TG samples of CFA-treated *Tac4^-/-^* and WT mice. Rows represent genes and columns represent TG samples (*n* = 3 in each group). Normalized gene counts data are shown as row-wise z-scores (scale is shown on legend). Rows and columns were hierarchically clustered using Pearson correlation distance measure and average method. Distances are shown as dendrograms. References for the functional annotations: *Bcas1* [64] *Pttg1* [65] *Brd4* [66,67] *Bcl2a1a* [68] *Ppp3ca* [69] *F3* [70,71] *Grn* [72,73] *Lmna* [74] *Tmc7* [75,76] *Reln* [77] *Fgf13* [78,79,80,81] *Cdc25a* [82] *Il1r2* [83] *Plxdc2* [84] *Tmem100* [85,86] *Fancd2* [87] *Gpr62* [88,89] *Bcl2l1* [90] *Kmt2c* [91] *Slfn8* [92,93] *Cflar* [94].

**Figure 8:**
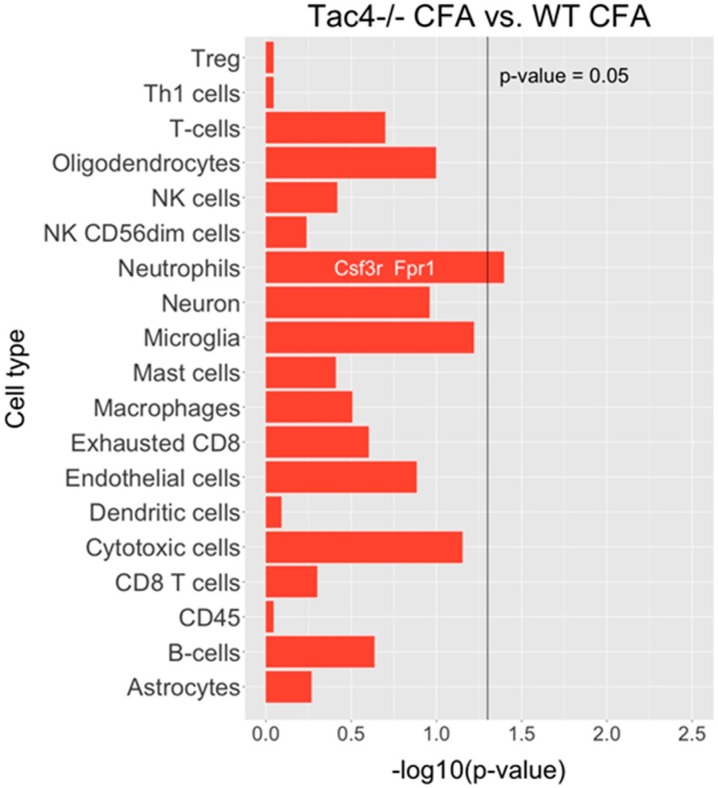
Barplots of *p*-values for correlation of cell-type-specific gene expressions as compared between CFA-treated *Tac4^-/-^* and WT mice. *p*-values are -log_10_ transformed.

Nanostring results revealed differentially expressed genes, as well as statistically significant correlations of cell-type-specific gene expressions. In TG samples of saline-treated *Tac4^-/-^* mice, 15 genes were found to be differentially expressed with a *p*-value threshold of 0.05 when compared to saline-treated WT mice. Nine genes were upregulated, six were downregulated in the *Tac4^-/-^* TG samples (Figure 5). There were gene expression differences in saline-treated (non-inflamed control) animals in case of genetic lack of HK-1 in the KO mice in comparison with WTs. Changes in microglia/macrophage and cytotoxic cell-specific genes were significantly correlated when saline-treated *Tac4^-/-^* mice were compared to saline-treated WTs (Figure 6). In TG samples of CFA-treated *Tac4^-/-^* mice, 22 genes were found to be differentially expressed with a *p*-value threshold of 0.05 when compared to CFA-treated WT animals. Thirteen genes were upregulated, 9 were downregulated in the *Tac4^-/-^* TG samples (Figure 7). Changes in genes specific to neutrophil granulocytes were significantly correlated when CFA-treated *Tac4^-/-^* mice were compared to CFA-treated WTs (Figure 8). For a further description of other comparisons between groups, see Supplementary Material (Supplementary Figures S4–S7).

## 3. Discussion

In the present study, we confirmed the presence of *Tac4* mRNA in the TG and established its upregulation in response to orofacial inflammation. While it had been previously shown that HK-1 was expressed widely in the nervous system including the brain, spinal cord, dorsal root ganglia, brain stem and the TG [34,39], this is the first study to assess the changes of *Tac4* expression alterations under pathological conditions. HK-1 had only been detected in small and medium-size neurons by immunohistochemistry in the rat TG [39], but in the present study, we showed that besides the neuronal expression, *Tac4* mRNA was also expressed in satellite glial cells of the TG. More importantly, significant inflammation-related upregulation of *Tac4* was shown in both neurons and satellite glial cells. Based on our facial mechanonociceptive threshold measurements and the qPCR results in the rat, we showed that *Tac4* upregulation occurred parallel to the development of allodynia, which suggests its potential role in the sensitization process. Although we provide evidence for expression changes only at mRNA level, which is a clear limitation of the study, the concomitant behavioral alterations suggest that the protein products of the examined mRNAs were also affected. Without the availability of specific and sensitive antibody against HK-1, we cannot further confirm it experimentally. The concomitant upregulation of HK-1 in trigeminal sensory neurons and SGCs is of particular importance, since an increasing amount of evidence points to the importance of neuron-glia crosstalk in chronic pain conditions, including orofacial pain [2,6,95,96,97,98]. Other neuropeptides released from peptidergic sensory neurons have already been suggested to have a prominent role in glial cell activation during sensitization. Among these, CGRP, which has a clinically proven role in migraine headaches [99], appears to be a key mediator of the neuron-glia interactions in the TG [4,100,101]. HK-1 could be a neuropeptide participating in two-way communication between sensory neurons and satellite glial cells. Moreover, we have also provided evidence on a possible physiological role of HK-1 in the trigeminal system by showing that there is a baseline difference in the expression of genes associated with glial cell activity in both the TG and the TNC. 

Previous studies by our and other groups have established that HK-1 contributes to the development of hyperalgesia in both acute and chronic pain models. HK-1 can elicit pain when injected intrathecally [34,43], and *Tac4^-/-^* mice have reduced nocifensive behavior in chemically-induced pain and suppressed hyperalgesia/allodynia in chronic inflammatory and neuropathic pain models [41,102]. In inflammatory pain and arthritis, a pro-inflammatory component is also likely to contribute to its action, since HK-1 expression was described in the immune system as well [30,33,103] and *Tac4* deficiency also alleviated experimental lung inflammation [42]. On the other hand, HK-1 was shown to have a direct role in central nociceptive sensitization, as spinal microglia and astrocyte activation was also attenuated in *Tac4^-/-^* mice after nerve injury [41]. HK-1 is a potent NK-1 receptor agonist [34], and most of its effects can be explained by NK-1 receptor activation. However, several lines of evidence point to a different mechanism of action compared to SP, and even a yet unknown target. This is supported by the observations that the phenotype of *Tac4^-/-^* deficient mice in chronic pain models is different from SP or NK-1 receptor-deficient mice and an opposite phenotype was also described in models of anxiety and depression as well [40]. 

We also investigated the contribution of HK-1 in trigeminal sensitization by adapting the CFA-induced orofacial pain model to mice to compare the allodynia and the changes of neuronal and glial activation markers between *Tac4^-/-^* and wild-type mice. In parallel, we confirmed the upregulation of *Tac4* in mice after inflammation by the ultrasensitive RNAscope technology [104]. However, RT-qPCR could detect neither basal nor upregulated *Tac4* in mice, probably owing to a low expression and the small tissue volume. Lamentably, we could not unequivocally show that orofacial allodynia was attenuated in *Tac4^-/-^* mice in the present study (Supplementary Figure S1a). The lack of clear behavioral functional data for the *Tac4^-/^*^-^ phenotype in this model is another limitation of our study. This technical issue is likely the light restraint that had to be used to measure the von Frey threshold of the face, which appeared to be an important stress factor. In contrast with rats, mice do not adapt well to repeated handling [105], therefore we tried to limit the handling and the number of repeated measurements to the minimum. Despite this, the mice were probably too stressed during the experiment, which could be one of the reasons that the mechanical thresholds of both saline and CFA-treated animals decreased from the baseline. It is also worth mentioning that based on our unpublished observations already the baseline von Frey threshold values of C57Bl/6 mice were very low compared to the threshold of NMRI mice, which usually show lower anxiety level in our experience. Stress-induced hyperalgesia is a well-known phenomenon which is both detectable in humans and animal models [106]. Other researchers use special restrainers or cages to measure the orofacial thresholds in mice to overcome the challenges of handling-induced stress [107,108]. We have previously used the technique in NMRI mice [109], but in our experience C57Bl/6 mice tolerated this type of restraint less compared to the light manual handling. Changes in spontaneous behavior measured in the open field test corroborated previous results of our group regarding the possible role of HK-1 in mediating anxiolytic actions [40], but we could not detect any pain-induced reduction of spontaneous activity either (Supplementary Figure S1b).

Our previous results in the orofacial inflammation model showed that in rats the mRNA levels of neuronal and glial activation markers changed parallel with the mechanical hyperalgesia in the TG, TNC and PBMCs [49]. In contrast with rats, we could not unequivocally reproduce the results of the activation marker changes in the mice, either. As mentioned before, there was already a difference in the baseline expression of glial activation markers in intact mice between the two genotypes. In the intact TG and TNC of *Tac4^-/^*^-^ mice, there was a lower expression of the microglia/macrophage marker *Iba1* and the SGC/astrocyte marker *Gfap* was also expressed at a significantly lower level in all the sampled tissues of *Tac4^-/^*^-^ mice. The basal difference in activity is not entirely surprising since macrophages are known to express both HK-1 and the NK-1 receptor and the *Tac4* mRNA was also detected in cultured microglia [36]. Likewise, previous data also revealed that astrocytes and microglia in the brain express NK-1 receptors [110] and NK-1 receptor antagonist treatment could reverse opioid withdrawal-induced astrocyte and microglia activation [111]. All activation markers increased significantly in both saline- and CFA-treated groups, which is most probably a consequence of the injection procedure and the previously mentioned restraint-induced stress during the von Frey threshold measurements. Both saline- and CFA-treated *Tac4^-/-^* mice had significantly lower levels of *FosB* and *Gfap* upregulation compared to their respective WT counterparts. The marked difference in *Gfap* between WT and *Tac4^-/-^* mice could also suggest that HK-1 plays a role in the activation of SGCs and astrocytes during stress. Similarly, NK-1 receptor upregulation in astrocytes was linked to stress-induced hyperalgesia and gastrointestinal motility disorders [112]. Nevertheless, compensatory changes secondary to *Tac4* deletion (e.g., expression changes of *Tac1*) cannot be excluded as factors responsible for the observed differences. 

Analysis of the Nanostring neuroinflammation panel kit data also revealed that there were a number of differentially expressed genes between saline- or CFA-treated wild-type and *Tac4^-/-^* mice. To our knowledge, this is the first such analysis in the TG to investigate neuroinflammation in a pain model. The cell-type-specific profiling in the comparison of saline-treated wild-type and *Tac4^-/-^* animals showed that microglia and cytotoxic cell-related genes were regulated in a significantly different extent compared to other cell types involved in inflammatory processes. As we discussed before, the effect of HK-1 deficiency on expression levels of macrophage/microglia-related genes is expected due to the previously described distribution of both the peptide and the NK-1 receptor. However, looking at individual differentially regulated genes, the functions of SGCs are also likely to be affected. The most interesting finding among these genes is the downregulation of *Kcnj10*, encoding the inwardly rectifying potassium channel subunit K_ir_4.1 in SGCs which has been linked to inflammatory sensitization [63] and pain modulation by GABA_B_ receptors [113]. Comparison of the cell-type-specific profiling of differentially expressed genes between CFA-treated wild-type and *Tac4^-/-^* mice only yielded a significant result in neutrophils. However, in the list of differentially expressed genes, we have found various genes related to macrophage/microglia activity, neuronal transmission, as well as genes mediating immune cell activation, such as calcineurin A or members of the Bcl-2 protein family, regulating apoptosis. Further validation of selected genes should be performed to confirm their importance in the effects of HK-1 in the TG.

In summary, we conclude that HK-1 released from sensory neurons and satellite glial cells may contribute to inflammatory processes and nociceptive sensitization underlying orofacial pain. HK-1 is suggested to have a role in mediating neuron-glia interactions both under physiological and inflammatory conditions. These results open interesting novel perspectives to identify the role and mechanisms of action of HK-1 in neuroinflammation in other models of trigeminal sensitization and to determine its potential clinical relevance. 

## 4. Materials and Methods 

### 4.1. Animals

Experiments were performed on male Wistar rats (Toxi-Coop, Hungary) weighing between 200–300 g, as well as on male, 8–12 week-old C57Bl/6 and *Tac4*-deficient (*Tac4^-/-^*) mice weighing between 20–25 g. The original breeding pairs of the *Tac4^-/-^* mice were generated as previously described [114]. Transgenic mice were generated on a C57Bl/6 background and backcrossed to homozygosity for >5 generations before using C57Bl/6 mice as controls, purchased from Charles River (Sulzfeld, Germany). Animals were kept under standard light-dark cycle (12-h light/dark cycle) and temperature (22 ± 2 °C) conditions. Food and water were provided ad libitum, in the Animal House of the Department of Pharmacology and Pharmacotherapy of the University of Pécs. All procedures were approved by the National Ethics Committee on Animal Research of Hungary on 1 Aug 2017 (license No.: BA02/2000-51/2017 issued on 22 Aug 2017 by the Government Office of Baranya County, Hungary) and were performed according to the European legislation (Directive 2010/63/EU) and Hungarian Government regulation (40/2013., II. 14.) on the protection of animals used for scientific purposes.

### 4.2. CFA Injection

Orofacial inflammation in mice was induced by bilateral s.c. injection of 10–10 μL complete Freund’s adjuvant (CFA; Sigma-Aldrich, Saint Louis, MI, USA; killed Mycobacteria suspended in paraffin oil; 1 mg/mL) into the whisker pad under i.p. ketamine (100 mg/kg) and xylazine (5 mg/kg) anaesthesia. In the case of rats, unilateral injection of 50 μL CFA was used, as previously described [49]. Control groups received the same volume of saline injection in both cases. We randomized the treatment of animals within each cage, ensuring a similar sample size for each treatment group.

### 4.3. Orofacial Pain Sensitivity Tested with von Frey filaments

A set of calibrated nylon monofilaments (Stoelting, Wood Dale, Illinois, USA) was used to perform measurements before and after CFA/saline injection on all animals. Increasing strengths (rats: 0.8–12 g, mice: 0.0075–1 g) were used to measure facial mechanosensitivity. The mechanonociceptive threshold was defined as the lowest force evoking at least 2 withdrawal responses (face stroking with the forepaw or head shaking) out of 5 stimulations according to our previous paper [49]. The experimenter was not blinded for these measurements as the visible inflammatory oedema of the whisker pad cannot be hidden during the in vivo experiments.

### 4.4. Sample Collection

For RT-qPCR analysis, TG and TNC tissue and PBMC samples were collected from animals on day 1, 3, and 7 after receiving s.c. CFA injection and following behavioral tests. Animals were anaesthetized with pentobarbital (rats: 50 mg/kg and mice 70 mg/kg i.p.) and sacrificed by exsanguination. TGs and TNCs were excised and snap-frozen in liquid nitrogen. 

For RNAscope, animals were transcardially perfused with 0.01 M phosphate-buffered saline (PBS; pH 7.6) followed by 4% paraformaldehyde solution on day 3 after CFA or saline injections. TGs were postfixed for 24 h at room temperature, rinsed in PBS, dehydrated, and embedded in paraffin using standard procedures. 5 µm sections were cut using a sliding microtome (HM 430 Thermo Fisher Scientific, altham, MA, USA). 

TG samples for Nanostring analysis were snap-frozen on day 3 and stored at −80 °C until use. RNAscope and Nanostring analysis were performed on samples derived from animals not involved in behavioral studies. Samples were processed in a blinded manner.

### 4.5. RNAscope In Situ Hybridization (ISH)

RNAscope assay was performed on 5 µm thick longitudinal TG sections using RNAscope Multiplex Fluorescent Reagent Kit v2 (ACD, Hayward, CA, USA) according to the manufacturer’s instructions. Briefly, tissue sections were baked, deparaffinized and H_2_O_2_-blocked, boiled, pretreated with Protease Plus and hybridized with mouse *Tac4, Kcnn3, Rbfox3,* mouse 3-plex positive and negative control probes. Signal amplification and channel development were applied sequentially. Nuclei were counterstained with 4′,6-diamidino-2-phenylindole (DAPI) and mounted with ProLong Glass Antifade Mountant for confocal imaging. Probes, applied dilutions of fluorophores are listed in Supplementary Table S1. Fluorescent images were acquired using an Olympus Fluoview FV-1000 laser scanning confocal microscope (Olympus, Tokyo, Japan) and Fluo-View FV-1000S-IX81 image acquisition software system. The confocal aperture was set to 80 µm. The analogue sequential scanning was performed using a 40× objective lens (NA: 0.75). The optical thickness was set to 1 μm and the resolution was 1024 × 1024 pixel. The excitation time was set to 4 µs per pixel. Virtual colours were selected to depict fluorescent signals: blue for DAPI, green for fluorescein (*Polr2a*), red for Cyanine 3 (*Tac4* and *Ppib*) and white for Cyanine 5 (*Ubc*). Images of the respective four channels were stored both individually and superimposed to evaluate the co-localization of fluorescent signals. Basal and elevated *Tac4* expression levels were analyzed semi-quantitatively using ImageJ software according to the manufacturer’s guideline in a blinded-manner. On rat TG, *Tac4* transcripts were quantified on *Tac4*-positive neurons and SGCs from CFA (neuron, *n* = 120; SGC, *n* = 69)-and saline (neuron, *n* = 68 SGC, *n* = 52)-treated animals, *n* = 4–6 rats/group. Similarly, on mouse TG, *Tac4* transcripts were quantified on *Tac4*-positive neurons and SGCs from CFA (neuron, *n* = 119; SGC, *n* = 54)-and saline (neuron, *n* = 71; SGC, *n* = 38)-treated animals, *n* = 4–5 mice/group.

### 4.6. Mononuclear Cell Separation from Peripheral Blood

PBMCs were obtained by Ficoll-PaquePREMIUM (GE Healthcare, Budapest, Hungary) standard density gradient centrifugation method [49]. Briefly, the mixture of fresh anticoagulant-treated blood, pooled from 3 individual mice and an equal volume of balanced salt solution was carefully overlaid on Ficoll-PaquePREMIUM and centrifuged. The mononuclear layer was transferred into a new tube and washed twice with the salt solution. The supernatant was discarded, the cells were resuspended in TRI Reagent (Zymo Research, Irvine, CA, USA) and stored at −80 °C until use.

### 4.7. Quantitative Real-Time PCR (RT-qPCR)

Total RNA purification, transcription and RT-qPCR from rat samples were performed exactly as described previously [49]. In the case of samples from mice, the same protocol was used with minor modifications as follows. TRI Reagent manufacturer’s (Zymo Research, Irvine, CA, USA) protocol was followed up to the step of acquiring the aqueous phase. Further RNA purification was made from the aqueous phase using the Direct-zol RNA MiniPrep kit (Zymo Research, Irvine, CA, USA) according to the manufacturer’s protocol. Nanodrop ND-1000 Spectrophotometer V3.5 (Nano-Drop Technologies, Inc., Wilmington, DE, USA) was used to define the quantity and purity of the extracted RNA. Total RNA was reverse transcribed using Maxima First Strand cDNA Synthesis Kit (ThermoScientific, Santa Clara, CA, USA), PCR amplification was performed using SensiFast SYBR Lo-ROX Kit (Bioline, Taunton, MA, USA). From the enlisted reference genes: glyceraldehyde 3-phosphate dehydrogenase (*Gapdh*), hypoxanthine phosphoribosyltransferase 1 (*Hprt1*), beta-2-microglobulin (*β2m*) and Peptidyl-prolyl cis-trans isomerase (*Ppia*), after transcripts were detected in all samples, *Ppia* (TG) and *Ppia*, *Gapdh* (PBMCs, TNC) were chosen as internal controls. Primers of similar efficiencies were used and 2^−ΔΔCq^ fold change values were calculated. All the primers used for RT-qPCR are listed in Supplementary Table S2. 

### 4.8. Nanostring

NanoString nCounter^®^ technology (NanoString Technologies, Seattle, WA, USA) was used, for expression profiling of RNA isolated from TG, according to the manufacturer’s instructions. Mouse Neuroinflammation Panel was composed of probes for 770 genes related to immunity and inflammation, neurobiology and neuropathology. 

Total RNA isolation and purification from mouse TG samples were performed using TRI Reagent and Direct-zol RNA MiniPrep kit as previously described, performing column DNase treatment as well. The RNA quality and quantity were measured using Bioanalyzer 2100 (Agilent, Santa Clara, CA, USA), Qubit Fluorometer Fluorescence Qubit 3.0 (ThermoFisher Scientific, Waltham, MA, USA) and Nanodrop ND-1000 Spectrophotometer V3.5 (Nano-Drop Technologies, Inc., Wilmington, DE, USA). Only samples with an RNA integrity number RIN >8.1 and 260/280 ratios of ∼2.0 were used for further analysis.

The RNA samples (25 ng of each) were processed using Mus musculus Neuroinflammation panel v1.0 according to the manufacturer instructions (user manual MAN-10023-11) on NanoString SPRINT Profiler instrument. Analysis of data was performed using nCounter^®^ Advanced Analysis plugin v2.0.115 for the nSolver Analysis Software v4.0.70 with the ProbeAnnotations_NS_Mm_NeuroInflam_v1.0 file provided by NanoString, using default settings. Briefly, raw data with gene counts lower than 50 were removed, suitable reference genes were evaluated using geNorm pairwise variation statistic, gene count data were normalized, differentially expressed genes were determined and cell type profiling was performed for each comparison.

Differentially expressed genes were plotted on heat maps where rows represent genes and columns represent TG samples. Normalized gene counts data are shown as row-wise z-scores (scale is shown on legend). Rows and columns were hierarchically clustered using Pearson correlation distance measure and average method. Distances are shown as dendrograms. Clustering and heat map generation were performed using the R language version 3.6.1 and the pheatmap package v1.0.12.

### 4.9. Statistical Analysis

GraphPad Prism software (GraphPad Software, Inc., La Jolla, CA, USA) was used for the statistical analysis of behavioral data, RT-qPCR and RNAscope quantification. After testing datasets for normal distribution, two-way analysis of variance (ANOVA) with repeated measures followed by Tukey’s multiple comparison tests was performed for time-matching samples (behavioral studies). One-way or two-way ANOVA followed by Tukey’s multiple comparison tests was used for RT-qPCR data. Student’s t-test for unpaired samples was preferred for RNAscope analysis to compare saline- and CFA-treated tissues. Sample numbers for each experiment are listed in Supplementary Table S3. Results are plotted as the mean ± standard error of the mean (SEM). Probability values *p* ≤ 0.05 were accepted as significant in all tests. 

Analysis of NanoString data was performed using nCounter^®^ Advanced Analysis Software v2.0.115. Differentially expressed genes were determined by applying log-linear model (linear regression) with a *p*-value threshold of 0.05. Correlation of cell-type marker genes was determined with a threshold of *p* < 0.05 for each comparison. 

## Supplementary Materials

Supplementary Materials can be found at https://www.mdpi.com/1422-0067/21/8/2938/s1.

## Abbreviations

CFA	Complete Freund’s Adjuvant
CGRP	Calcitonin gene-related peptide
GFAP	Glial fibrillary acid protein
HK-1	Hemokinin-1
IBA1	Ionized calcium-binding adaptor molecule 1
PBMCs	Peripheral blood mononuclear cells
SGC	Satellite glial cell
SP	Substance P
TG	Trigeminal Ganglion
TNC	Trigeminal Nucleus Caudalis
