# Supplementary material for: Hemokinin-1 Gene Expression Is Upregulated in Trigeminal Ganglia in an Inflammatory Orofacial Pain Model: Potential Role in Peripheral Sensitization
Source: Int J Mol Sci. 2020 Apr 22;21(8):2938. doi: 10.3390/ijms21082938 (PMC7215309; doi:10.3390/ijms21082938)
Supplement: Supplementary file 1 [file ijms-21-02938-s001.pdf]

International Journal of
*Molecular Sciences*

**Supplementary Materials for *Hemokinin-1 Gene Expression is Upregulated in Trigeminal Ganglia in an Inflammatory Orofacial Pain Model: Potential Role in Peripheral Sensitization***

## 1. Supplementary Results

### *1.1. Behavioural tests in Tac4$^{-/-}$ gene-deficient mice*

CFA administration caused significantly decreased facial mechanonociceptive threshold 1 and 3 days after injection in both wild-type (WT) and *Tac4*$^{-/-}$ mice. However, saline injection caused similar, although non-significant changes. No significant changes were observed between the thresholds of WT and *Tac4*$^{-/-}$ mice. No significant change in spontaneous behavior was seen as a consequence of CFA injection, although *Tac4*$^{-/-}$ mice spent less time in the centre zone of the open-field box, suggesting an anxiety-like behavior compared to their littermates (Supplementary Figure 1).

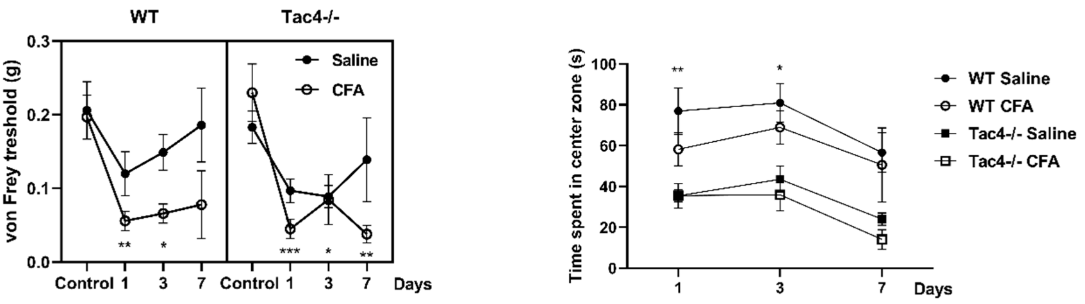


(**a**) (**b**)

**Supplementary Figure 1.** (**a**) Changes in von Frey threshold before and 1, 3, 7 days after CFA (10 μl s. c. complete Freund's adjuvant) inflammation. Mean of the orofacial thresholds in both sides were calculated. Data are means ± S.E.M. (n = 7–18). Asterisks denote statistically significant differences between control and saline/CFA-treated groups (*$p \leq 0.05$, **$p \leq 0.01$, ***$p \leq 0.001$) as analyzed by two-way ANOVA followed by Tukey's multiple comparison tests; (**b**) Changes in spontaneous behavior measured in the open field test. Data are means ± S.E.M. (n = 3–15; WT vs. *Tac4*$^{-/-}$; *$p < 0.05$, **$p \leq 0.01$, two-way ANOVA followed by Tukey's multiple comparison tests).

### 1.2. RNAscope

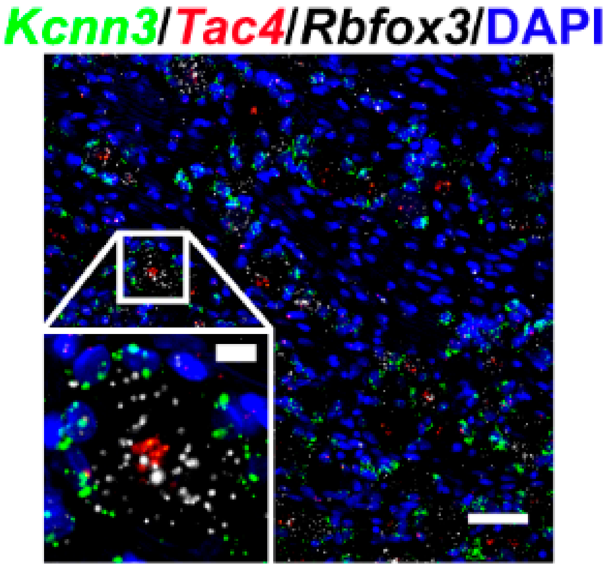


**Supplementary Figure 2.** Representative confocal image of rat longitudinal TG section. RNAscope probes specific to *Kcnn3* (in green encoding SK3) to detect SGCs, and to *Rbfox3* (in white encoding NeuN) to visualize sensory neurons were used to colocalize with *Tac4* mRNA (in red). Scale bar: 50 µm, inset scale bar 10 µm.

### 1.3. RNAscope technical controls

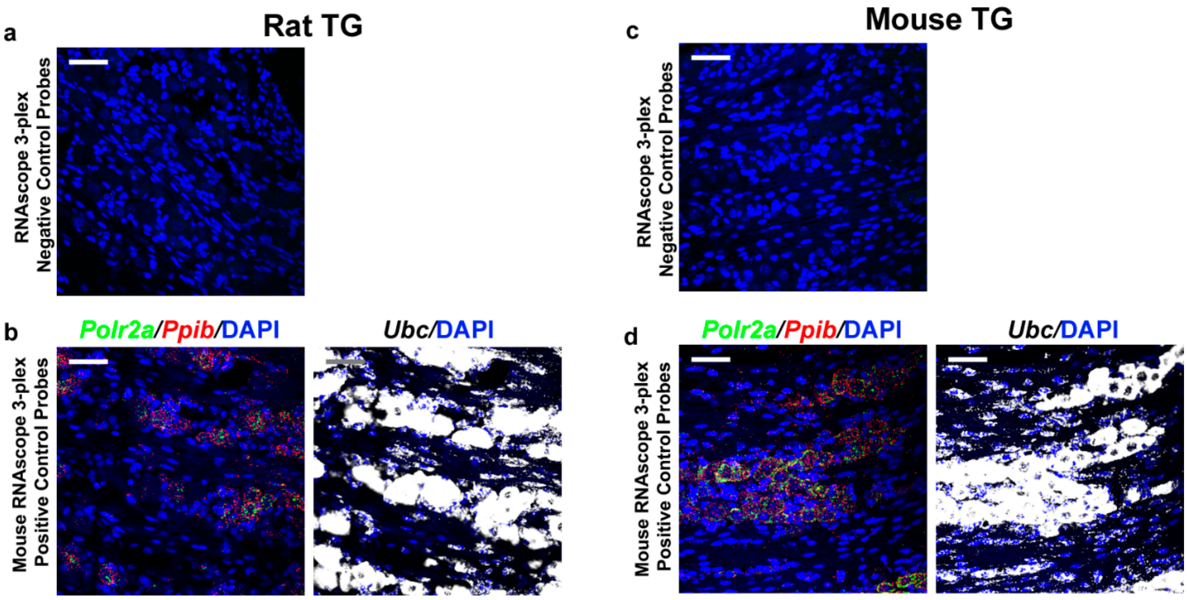


**Supplementary Figure 3.** RNAscope control conditions performed on saline-injected rat and mouse longitudinal TG sections counterstained with DAPI. **(a)** Representative confocal image of 3-plex negative control probes specific to the bacterial *dabP* gene giving no specific signal on rat TG; **(b)** Representative confocal images of 3-plex positive control probes specific to mouse *Polr2a* (in green), *Ppib* (in red) and *Ubc* (in white) mRNA targets on rat TG; **(c)** Representative confocal image of 3-plex negative control probes specific to the bacterial *dabP* gene giving no specific signal on mouse TG; **(d)** Representative confocal images of 3-plex positive control probes specific to mouse *Polr2a* (in green), *Ppib* (in red) and *Ubc* (in white) mRNA targets on mouse TG. Scale bar: 50 µm. Note that sensitivity of mouse 3-plex positive control probes applied on rat tissue samples reflects on the highly conserved mouse and rat genome.

## *1.3. Nanostring*

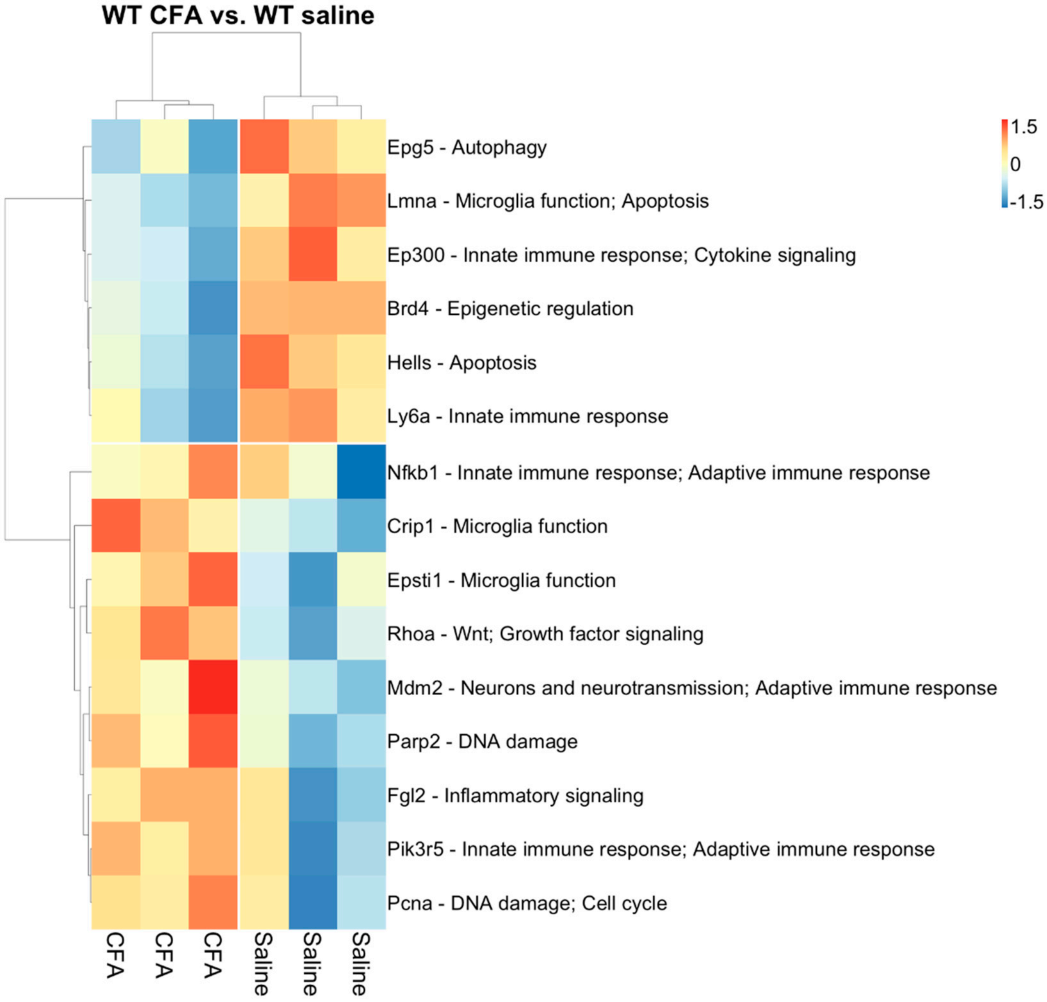


**Supplementary Figure 4.** Heat map representation of the differentially expressed genes with annotations between TG samples of CFA and saline-treated WT mice. Rows represent genes and columns represent TG samples (n = 3). Normalized gene counts data are shown as row-wise z-scores (scale is shown on legend). Rows and columns were hierarchically clustered using Pearson correlation distance measure and average method. Distances are shown as dendrograms.

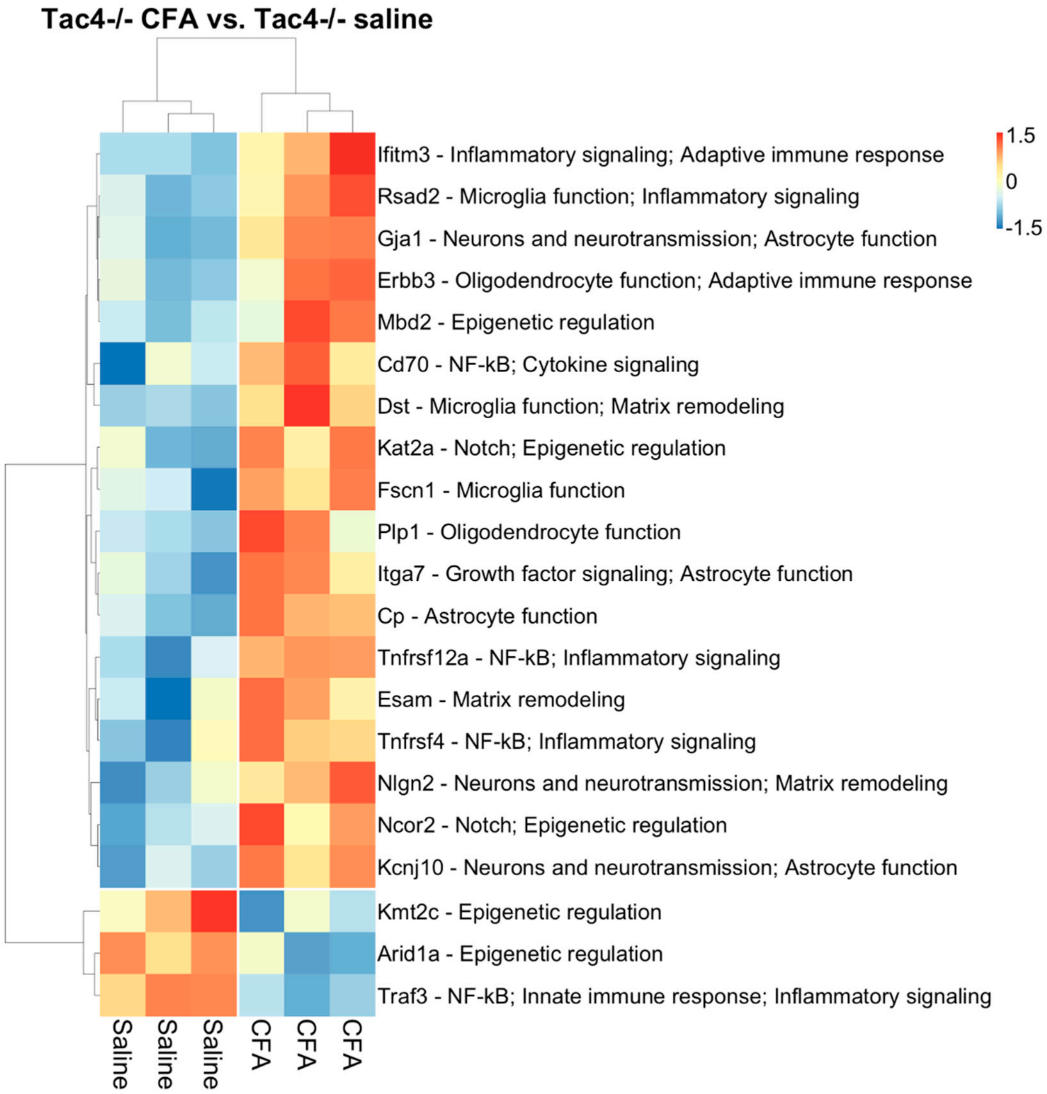


**Supplementary Figure 5.** Heat map representation of the differentially expressed genes with annotations between TG samples of CFA- and saline-treated *Tac4*$^{-/-}$ mice. Rows represent genes and columns represent TG samples (n = 3). Normalized gene counts data are shown as row-wise z-scores (scale is shown on legend). Rows and columns were hierarchically clustered using Pearson correlation distance measure and average method. Distances are shown as dendrograms.

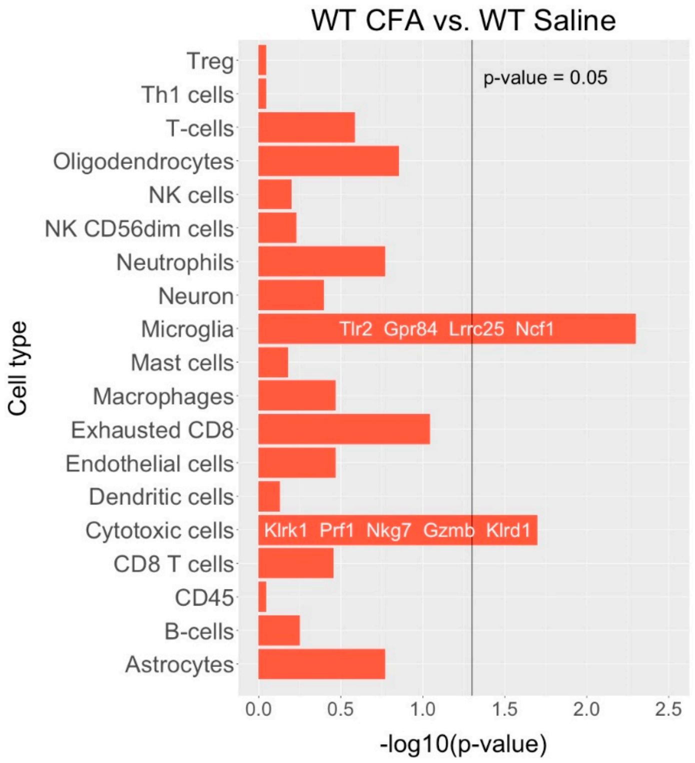


**Supplementary Figure 6.** Barplots of p-values for correlation of cell-type-specific gene expression as compared between CFA- and saline-treated WT mice. Barplots of p-values across cell types. p-values are -log10 transformed. Statistically significant cell types ($p < 0.05$) are shown.

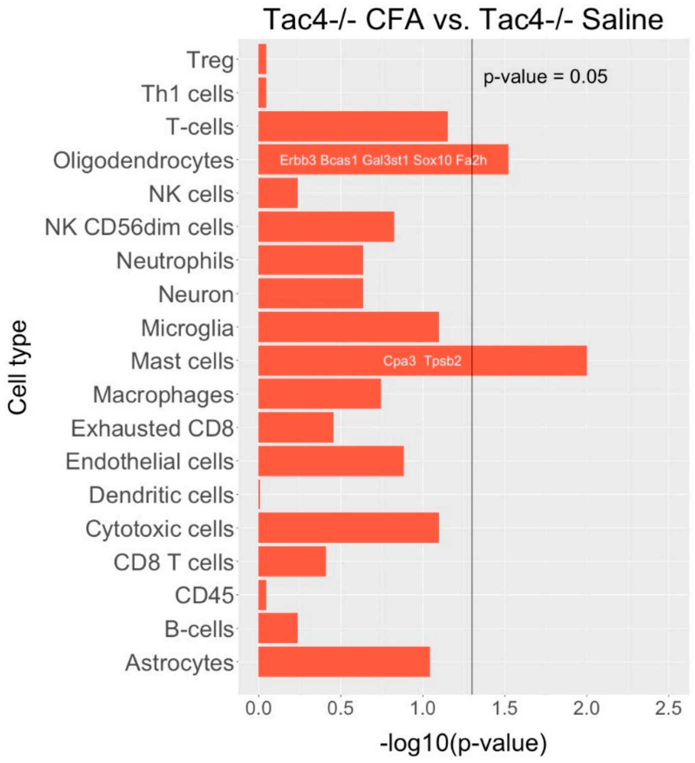


**Supplementary Figure 7.** Barplots of p-values for correlation of cell-type-specific gene expression as compared between CFA- and saline-treated *Tac4*$^{-/-}$ mice. Barplots of p-values across cell types. p-values are -log10 transformed. Statistically significant cell types ($p < 0.05$) are shown.

## 2. Supplementary Materials and Methods

### 2.1. Open Field test

The open-field test was used to assay spontaneous activity and anxiety levels of mice. The mice were placed into the same area of a box (60 cm x 40 cm), then their behavior was recorded for 10 min. Recorded videos were evaluated using Ethovision software (Noldus Information Technology, Wageningen, Netherlands).

### 2.2. RNAscope

**Supplementary Table 1.** Probes, applied dilutions of fluorophores

| Target | Catalog number | Fluorophores | Dilutions |
|---|---|---|---|
| *Tac4* | 449651-C2 | TSA Plus Cyanine 3 | 1:750 |
| *Kcnn3* | 427961 | TSA Plus Fluorescein | 1:750 |
| *Rbfox3* | 313311-C3 | TSA Plus Cyanine 5 | 1:750 |
| Mm-3-plex positive ctrl probes | 320881 | TSA Plus Fluorescein, Cyanine 3, 5 | 1:750 |
| 3-plex negative ctrl probes | 320871 | | |

## *2.3. Quantitative real-time PCR (RT-qPCR)*

**Supplementary Table 2.** Sequences of primers for real-time PCR

| Gene symbol | Species | Accession number | Forward primer sequence (F, 5' - 3')<br>Reverse primer sequence (R, 5' - 3') |
|---|---|---|---|
| *Aif1* (*Iba1*) | mouse | NM_001361501.1 | F: CAA CAA GCA ATT CCT CGA TGA TCC<br>R: CTC CAG CAT TCG CTT CAA GG |
| *β2m* | mouse | NM_009735.3 | F: TTC TGG TGC TTG TCT CAC TGA<br>R: CAG TAT GTT CGG CTT CCC ATT C |
| *FosB* | mouse | NM_001347586.1<br>NM_008036.2 | F: CGA GAA GAG ACA CTT ACC CCA<br>R: GTT TCC GCC TGA AGT CGA TCT |
| *Gapdh* | mouse | AY618199.1 | F: GTG GAG TCA TAC TGG AAC ATG TAG<br>R: AAT GGT GAA GGT CGG TGT G |
| *Gfap* | mouse | NM_010277.3 | F: CGG AGA CGC ATC ACC TCT G<br>R: TGG AGG AGT CAT TCG AGA CAA |
| *Hprt1* | mouse | NM_013556.2 | F: CCC CAA AAT GGT TAA GGT TGC<br>R: AAC AAA GTC TGG CCT GTA TCC |
| *Ppia* | mouse | NM_008907.2 | F: GAG CTG TTT GCA GAC AAA GTT<br>R: CCC TGG CAC ATG AAT CTT GG |
| *Tac4* | mouse | NM_053093.2 | F: CCG TGA ACC TGA AGG GAA T<br>R: CCC ATC AGA CCA TAG AAC TGG |
| *B2m* | rat | NM_012512 | F: CCC ACC CTC ATG GCT ACT TC<br>R: CCA CTT CAC TTC ACT CTG GCA |
| *Gapdh* | rat | NM_017008 | F: GTA ACC AGG CGT CCG ATA C<br>R: TCC TCT GCT CCT CCC TGT TC |
| *Hprt1* | rat | NM_012583 | F: GCT TTT CCA ACT TTC GCT GAT G<br>R: GGT GAA AAG GAC CTC TCG AAG |
| *Ppia* | rat | NM_017101 | F: CCA TTA TGG CGT GTG AAG TC<br>R: GCA GAC AAA GTT CCA AAG ACA G |
| *Tac4* | rat | NM_172328.2 | F: CTG TCC CCA GCA TCG AAC TT<br>R: CCA GCT GAT ACC CCG TTC TC |

**Supplementary Table 3.** A summary of sample numbers in each experiment. Samples for RT-qPCR were collected from animals participating in the behavioral measurements

| *Rat* | | | | |
|---|---|---|---|---|
| **Method** | **Treatment** | **Day 1** | **Day 3** | **Day 7** |
| **von Frey** | CFA | 16 | 10 | 5 |
| | saline | 6 | 6 | 6 |
| **RT-qPCR** | CFA | 3 | 3 | 3 |
| | saline | 3 | | |
| **RNAscope** | CFA | | 6 | |
| | saline | | 4 | |

| *Mouse* | | | | |
|---|---|---|---|---|
| **WT** | | | | |
| **Method** | **Treatment** | **Day 1** | **Day 3** | **Day 7** |
| **von Frey/Open field** | CFA | 18/13 | 18/13 | 8/3 |
| | saline | 16/13 | 16/13 | 7/4 |
| **RT-qPCR** | CFA | 5 | 10 | 3 |
| | saline | 5 | 9 | 4 |
| **RNAscope** | CFA | | 5 | |
| | saline | | 4 | |
| **Nanostring** | CFA | | 3 | |
| | saline | | 3 | |
| **Tac4 -/-** | | | | |
| **Method** | **Treatment** | **Day 1** | **Day 3** | **Day 7** |
| **von Frey /Open field** | CFA | 16/13 | 16/13 | 8/5 |
| | saline | 18/14 | 18/15 | 8/5 |
| **RT-qPCR** | CFA | 5 | 7 | 5 |
| | saline | 5 | 10 | 5 |
| **Nanostring** | CFA | | 3 | |
| | saline | | 3 | |
